# Immobilization of Microcystin by the Hydrogel–Biochar Composite to Enhance Biodegradation during Drinking Water Treatment

**DOI:** 10.1021/acsestwater.3c00240

**Published:** 2023-08-29

**Authors:** Lixun Zhang, Shengyin Tang, Sunny Jiang

**Affiliations:** Department of Civil and Environmental Engineering, University of California, Irvine, California 92697, United States

**Keywords:** microcystin-LR, selective adsorption, natural organic matter, lake water, computational calculation, adsorption−biodegradation

## Abstract

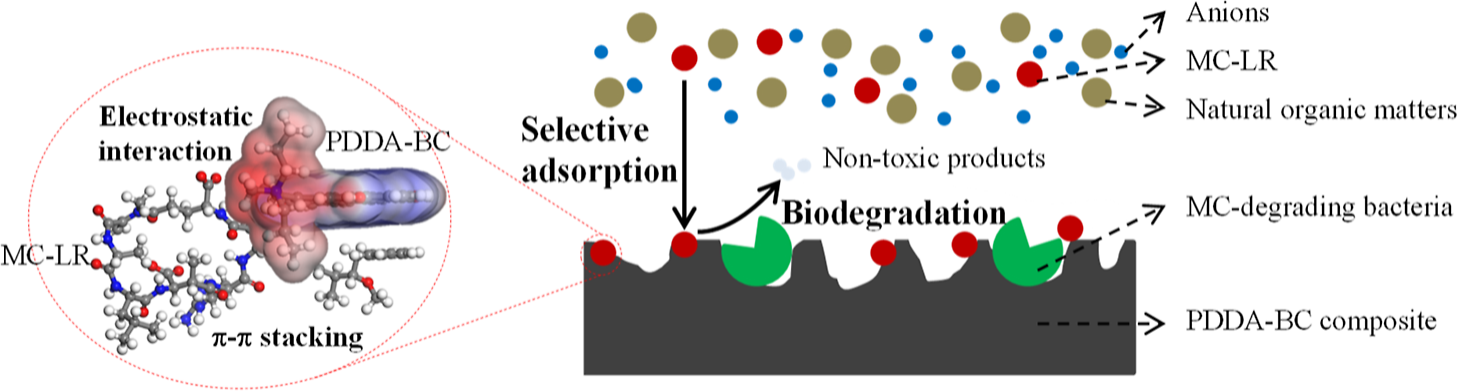

Microcystin-LR (MC-LR), the most common algal toxin in freshwater, poses an escalating threat to safe drinking water. This study aims to develop an engineered biofiltration system for water treatment, employing a composite of poly(diallyldimethylammonium chloride)–biochar (PDDA–BC) as a filtration medium. The objective is to capture MC-LR selectively and quickly from water, enabling subsequent biodegradation of toxin by bacteria embedded on the composite. The results showed that PDDA–BC exhibited a high selectivity in adsorbing MC-LR, even in the presence of competing natural organic matter and anions. The adsorption kinetics of MC-LR was faster, and capacity was greater compared to traditional adsorbents, achieving a capture rate of 98% for MC-LR (200 μg/L) within minutes to tens of minutes. Notably, the efficient adsorption of MC-LR was also observed in natural lake waters, underscoring the substantial potential of PDDA–BC for immobilizing MC-LR during biofiltration. Density functional theory calculations revealed that the synergetic effects of electrostatic interaction and π–π stacking predominantly contribute to the adsorption selectivity of MC-LR. Furthermore, experimental results validated that the combination of PDDA–BC with MC-degrading bacteria offered a promising and effective approach to achieve a sustainable removal of MC-LR through an “adsorption–biodegradation” process.

## Abstract 2

This study reports the selective and ultrafast removal of algal toxin MC-LR by a hydrogel−biochar composite and coupling with biodegradation.

## 1 Introduction

Harmful cyanobacterial blooms in surface water have become an increasing problem globally due to climate change and water eutrophication. Microcystins (MCs) are the most widely distributed and abundant toxins produced during cyanobacterial blooms. MC concentrations in freshwater used for drinking water production are often hundreds to thousands times greater than the World Health Organization (WHO) provisional guideline value of 1.0 μg/L.^1−3^ U.S. Environmental Protection Agency’s National Lakes Assessment (NLA) collected the MC data from lakes and reservoirs in the United States in the summers of 2007 and 2012 and found MC concentrations of up to 225 μg/L.^4^ MC concentration of 2153 μg/L was reported in Canadian lakes.^5^ Among all MC variants, microcystin-LR (MC-LR), a hepatotoxin, carcinogen, and reproductive toxicant,^6,7^ is the most common and toxic. It is critical to find an efficient and low-cost water treatment method to remove MC-LR for public health protection.

The bacterial biodegradation of MC-LR has the potential to be an environmentally friendly and cost-effective biotechnology that produces no harmful by-products.^8−11^ Natural bacterial strains or consortia that can effectively degrade MC-LR have been isolated from the natural water.^8−10^ However, biodegradation requires a minimum of 24 h retention time for the complete removal of the toxin, while the hydraulic retention time during biofiltration is much shorter.^12^ Therefore, the biodegradation is necessary to be facilitated by capturing toxins on the filtration media to increase the toxin retention time during biofiltration without slowing the water filtration efficiency.

A review of materials for potential immobilization of biotoxin indicates many natural adsorbents, such as activated carbon, graphene oxide, biochar (BC), silica, and clays, have been previously tested for MC-LR captivation.^13−16^ They indiscriminately adsorb the toxin along with natural organic matter (NOM), inorganic ions, and other water constituents.^17−20^ NOM is typically present at significantly higher concentrations than MC-LR in natural freshwater, leading to competition for adsorption sites. Due to the higher aromaticity compared to MC-LR, some NOM readily form bonds with the active sites of various adsorbents (e.g., activated carbon, graphene oxide, and BC) through π–π interactions. Moreover, many NOM are bigger in size than MC-LR, which block the mesopores (2–50 nm) of the adsorbents that are responsible for the MC-LR adsorption.^17,21^ Thus, the presence of NOM can greatly suppress the adsorption of MC-LR on the traditional adsorbents. In addition, since MC-LR mainly exists as a negatively charged form in the solution in pH range from 2.19 to 12.48,^22^ the presence of inorganic anions (such as Cl^–^ and SO_4_^2–^) may affect the electrostatic attraction of MC-LR on the adsorbents, reducing the adsorption efficiency. Improvements in both adsorption capacities and kinetics of these adsorbents (i.e., activated carbon and BC)^17,23,24^ have the potential to enhance their suitability as filtration media for the immobilization of MC-LR in practical applications.

The goal of this research is to develop functional biomaterials capable of selectively capturing MC-LR to enhance biodegradation during biofiltration. Our vision is for these biomaterials to replace sand as biofiltration media, effectively capturing MC-LR during water filtration. Additionally, the biomaterials will act as attachment substrates for MC-degrading bacteria, supporting bacteria growth and facilitating the efficient decomposition of MC-LR into non-toxic by-products.

Cationic hydrogel has gained attention in recent years as a strong adsorbent for anionic contaminants, such as perfluorinated alkyl substances,^25,26^ dyes,^27^ selenate and hexavalent chromium,^28^ fluoride,^29^ and phosphate^30^ because of its high selectivity, fast kinetics, and high capacity. However, pure hydrogels, such as poly(diallyldimethylammonium chloride), aka, PDDA, are mostly in liquid or gel form. Polymerized pure hydrogels have weak mechanical strength and low surface area and cannot be used as filtration media.^31^ BC can serve as the structure support for cationic hydrogels because of its advanced porous structure, high surface area, and excellent mechanical strength.^28,30^ In addition to mechanical support, BC also offers active sites (such as benzene ring and oxygen-containing groups) to capture MC-LR.^16,17^ The integration of cationic hydrogel and BC may enable a synergistic MC-LR immobilization mechanism, including electrostatic interactions, cation−π bonding, π–π stacking, and hydrogen bonding, to achieve selective and efficient captivation of MC-LR.

The objectives of the study are to: (1) synthesize and characterize hydrogel–BC composite; (2) evaluate the selectivity of MC-LR adsorption by the hydrogel–BC composite in the presence of competing NOM surrogates and anions; (3) investigate the adsorption kinetics and isotherms of MC-LR; (4) test the performance of hydrogel–BC for MC-LR adsorption in the natural water; (5) elucidate the binding mechanisms of MC-LR to the composite using density functional theory (DFT) modeling; and (6) explore the coupling of hydrogel–BC adsorption with specific MC-degrading bacteria for removal of MC-LR.

## 2 Materials and Methods

### 2.1 Synthesis and Characterization of Hydrogel–BC Composite

Pristine BC (Lewis Bamboo Inc., Alabama, United States) was grinded in a mortar and then sieved through a 100-mesh stainless steel sieve to prepare <150 μm BC particles. Cationic hydrogel, PDDA (20 wt % in water, 1.04 g/mL and 600–900 cP at 25 °C, Sigma-Aldrich, St. Louis, MO, United States) was used to synthesize hydrogel–BC composite. PDDA–BC composite was prepared by adding 4 g of sieved BC particles into 40 mL of 10 wt % of PDDA solution that was diluted using Milli-Q water. The mixture was continuously agitated for 24 h at room temperature for complete coating of PDDA on BC surface. Next, the solid was pelleted by centrifugation (Eppendorf 5810R, Enfield, CT, United States) at 4000 rpm, room temperature, then washed using Milli-Q water and pelleted again. This process was repeated for three times to remove un-adsorbed PDDA. The final product was dried overnight at 70 °C to a constant weight. Finally, the dry PDDA–BC composite was crushed and sieved through a 100-mesh stainless steel sieve and stored for further use.

The pore size distribution of the composite was determined by N_2_ adsorption–desorption experiments using Belsorp Max (MicrotracBEL, Japan). The surface morphology of PDDA–BC composite was visualized using an FEI Magellan 400 XHR scanning electron microscope (SEM, FEI Company, Hillsboro, OR). The surface chemical states of solid samples were determined by X-ray photoelectron spectroscopy (XPS) on a Kratos AXIS Supra spectrometer (Kratos Analytical, Kyoto, Japan) equipped with a dual anode Al/Ag monochromatic X-ray source.

### 2.2 Batch Adsorption Experiments

Batch experiments were performed in 25 mL glass bottles to examine PDDA–BC selective adsorption of MC-LR in the presence of ions and NOM. Cl^–^ and SO_4_^2–^ were used to represent inorganic anions, while Fisher Scientific humic acid (FSHA), Suwannee River humic acid (SRHA), and Suwannee River fulvic acid (SRFA) were used to represent NOM.^32,33^ Humic acid represents larger and more hydrophobic NOM than fulvic acid. The SRHA and SRFA were purified from the black water of Suwannee River, Georgia, and are sold by the International Humic Substances Society^34^ as a standard for natural organic research. FSHA is a sodium salt produced and sold by Fisher Scientific with 45–70% as humic acid according to the vendor. MC-LR (MilliporeSigma, Burlington, United States) stock solution was prepared in Milli-Q water. Each batch experiment contained 4 mg of PDDA–BC and a final concentration of 200 μg/L MC-LR in a 10 mL volume. The experimental groups included Milli-Q water only as control, Milli-Q water spiked with Cl^–^ or SO_4_^2–^ at 10, 50, 100, 200 mg/L, and Milli-Q water with FSHA at 1, 5, 10, 30 mg/L. The electrical conductivity of Milli-Q water spiked with 200 mg/L Cl^–^ and SO_4_^2–^ was 844 and 646 μs/cm, respectively. The mixtures were shaken on a thermostatic shaker (New Brunswick Scientific, United States) at room temperature and 180 rpm for 24 h. At the end of the adsorption, the mixture was filtered through a 0.22 μm pore-size PES syringe filter (MilliporeSigma) and the filtrate was used for MC-LR quantification. Effects of FSHA, SRHA, or SRFA (30 mg/L) on MC-LR (200 μg/L) removal at different pH (2–12) were further investigated to evaluate the pH-dependence of MC-LR adsorption by PDDA–BC. Solution pH was adjusted using 0.1 M of HCl or NaOH solution. The 200 μg/L initial MC-LR concentration was used in the experiments to represent the high end of the MC-LR concentration detected in the environment to ensure the treatment efficiency to meet the drinking water guideline.

Adsorption kinetics was also investigated at an initial MC-LR concentration of 200 μg/L and 0.4 g/L PDDA–BC at room temperature. The experiments were performed in Milli-Q water, 30 mg/L of FSHA solution, 200 mg/L of SO_4_^2–^ solution, and a lake water sample collected from San Joaquin Marsh & Sanctuary (33°39′35″ N, 117°50′43″ W) in Irvine, California. Sub-samples were collected for MC-LR analysis from each experimental group at pre-determined time intervals over a 3 or 24 h reaction time.

To determine the maximum adsorption capacity of PDDA–BC toward MC-LR in pure water and in lake water, batch isotherm experiments were performed using initial MC-LR concentrations ranging from 4.5 to 8 mg/L for adsorption to reach saturation. After a 24 h equilibrium, suspensions were filtered through a 0.22 μm pore-size PES syringe filter (MilliporeSigma) and collected for MC-LR analysis.

All experiments were conducted in triplicate. Solution pH in all experiments were not adjusted unless otherwise stated. The concentration of MC-LR was quantified on a Quattro Premier XE UPLC-MS/MS instrument (Waters, Milford, MA). An Acquity UPLC BEH C_18_ column (2.1 × 50 mm, 1.7 μm particle size) was used to separate the MC-LR. The column temperature was kept at 50 °C. The injection volume was 10 μL. The mobile phases consisted of 10 mM ammonium formate and 0.1% formic acid in water (solvent A), and 0.1% formic acid in acetonitrile (solvent B). The *m*/*z* values of parent → daughter ions were 995.5 → 135 for MC-LR and 825.4 → 135 for nodularin, respectively.

### 2.3 Computational Calculation

DFT simulation was used to explore the adsorption mechanisms of MC-LR onto the PDDA–BC. All geometry optimization and energy calculations were performed using the Dmol3 package in Materials Studio (Accelrys Software Inc., San Diego, United States). A graphene structure composed of seven aromatic rings and edge hydroxyls was used as the model of pristine BC.^35,36^ A quaternary ammonium group (N^+^) was introduced at the edge of the BC to present the structure of PDDA–BC based on the assumption that the hydrogel was attached to the surface of BC (Figure S1).^30,36,37^ The exchange–correlation function was described using the generalized gradient approximation Perdew–Burke–Ernzerhof. The core treatment was set as the DFT semi-core pseudopotential. The dual numerical polarization was selected as the basis set. The conductor-like screening model (COSMO) with a water permittivity of 78.54 was implemented to investigate the effect of water on the adsorption. The convergence tolerance of energy, force, and displacement were 1 × 10^–4^ Ha, 0.004 Ha/Å, and 0.005 Å, respectively. The adsorption energies were calculated by the following expression:
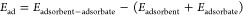
where, *E*_adsorbent–adsorbate_ is the total energy of the complex system after adsorption, *E*_adsorbent_ is the energy of adsorbent material, and *E*_adsorbate_ is the energy of adsorbate molecule.

### 2.4 Coupling PDDA–BC with *Sphingopyxis* sp. m6 for MC-LR Removal

Frozen *Sphingopyxis* sp. m6, an environmental MC-degrading bacterium that was isolated and characterized by Professor Pu’s lab (Southeast University, China)^9^ was activated in Luria Bertani (LB) medium. Single colonies were re-isolated on a LB agar plate at 30 °C. A single colony was then inoculated in 20 mL LB medium and incubated for 24 h. The fresh culture was centrifuged (4000 rpm, 10 min, 4 °C) to pellet the cells, followed by washing using M9 minimal medium (Na_2_HPO_4_·7H_2_O 12.8 g/L, KH_2_PO_4_ 3.0 g/L, NaCl 0.5 g/L, NH_4_Cl 1.0 g/L, NaNO_3_ 0.25 g/L, MgSO_4_ 0.002 g/L, and CaCl_2_ 0.001 g/L, electrical conductivity = 9.1 ms/cm). Finally, the washed bacterial pellet was suspended in the M9 medium, and the concentration of bacterial suspension was adjusted to OD_600_ = 0.40 ± 0.02.

Coupled adsorption–biodegradation experiments were performed in triplicate in a 100 mL conical flask. 3 g/L of PDDA–BC suspension and 35 mg/L of MC-LR stock solution were prepared using M9 medium. 20 mL of bacterial suspension was mixed with 4 mL of PDDA–BC suspension and incubated overnight at room temperature to allow bacteria attachment to the composite. Then 6 mL of MC-LR solution was added into the flask and the kinetics experiments were performed on a shaker at room temperature. Samples were collected at various time points (5 min, 1, 3, 5, 7, 10, 13, 18, and 24 h) for MC-LR analysis. Control experiments without the addition of PDDA–BC or *Sphingopyxis* sp. m6 were performed in M9 medium to examine the MC-LR removal by adsorption only or biodegradation only. The total number of bacteria in the mixture was determined by spreading the PDDA–BC–bacteria mixture on LB agar plates. The unattached bacteria were quantified by plating the filtrates after removing the solids by 5 μm PVDF membrane (MilliporeSigma). The solid samples on membrane were freeze-dried for SEM analysis. The contribution of adsorption and biodegradation to MC-LR removal was investigated using a generalized linear model (GLM).^38^ All the experiments were conducted in M9 media with electrical conductivity of 9.1 ms/cm and pH of 7.0.

## 3 Results and Discussion

### 3.1 Selective Captivation of MC-LR by Adsorption on PDDA–BC

**Figure 1:**
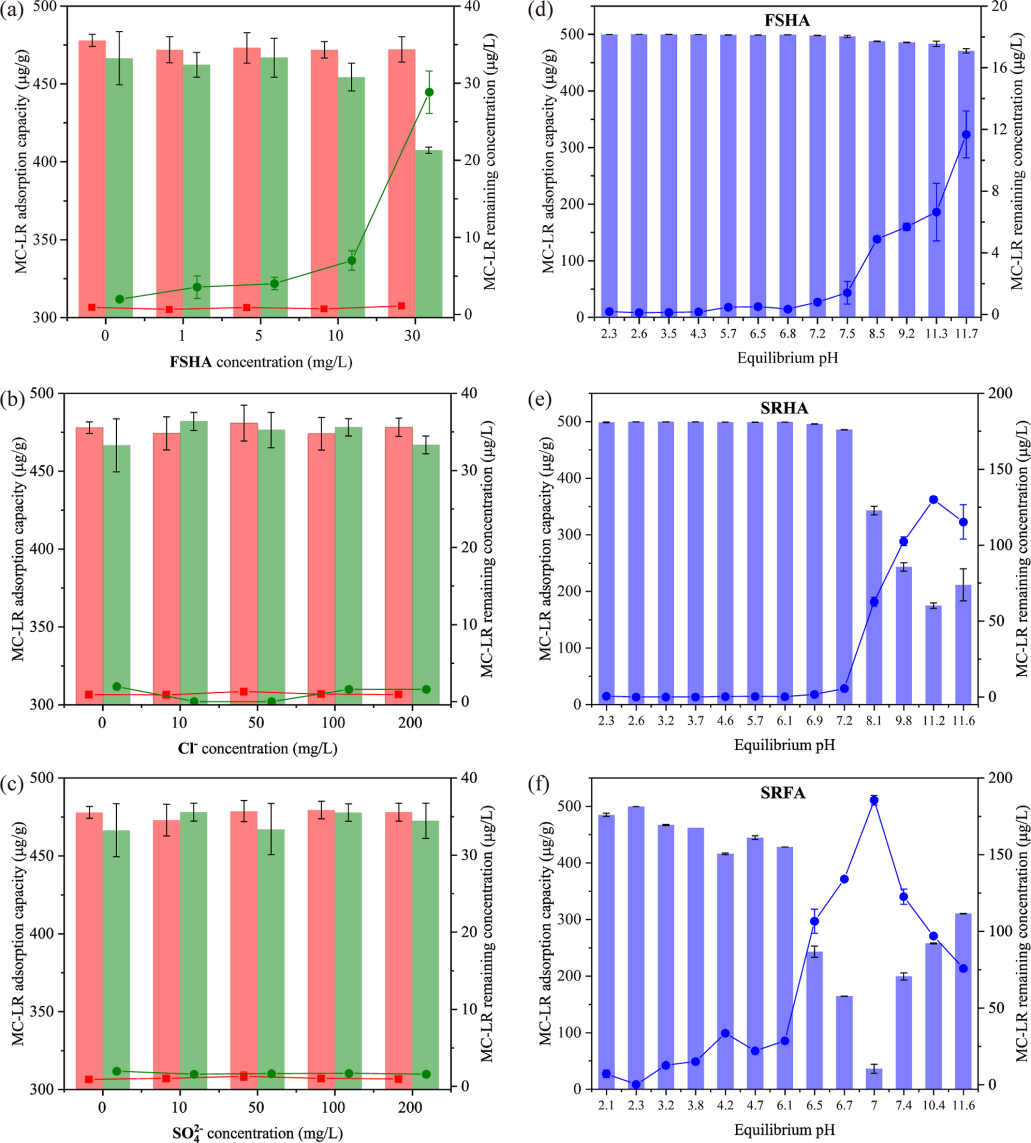
Effects of NOM and anions on the adsorption of MC-LR by PDDA–BC (red) and pristine BC (green) in the presence of (a) FSHA, (b) Cl^–^ ion, or (c) SO_4_^2–^ ion. Effects of pH on MC-LR adsorption by PDDA–BC (blue) in the presence of 30 mg/L of (d) FSHA, (e) SRHA, or (f) SRFA. The bar graphs represent adsorption capacity (left axis, μg/g), while the line graphs represent MC-LR remaining in solution (right axis, μg/L).

Effects of ions and NOM on MC-LR captivation by pristine BC and PDDA–BC are shown in Figure 1a–c. The results indicated that 200 μg/L of MC-LR was nearly completely adsorbed by PDDA–BC after 24 h in the presence of FSHA at concentrations of 0, 1, 5, 10, and 30 mg/L (Figure 1a). In comparison, the adsorption capacity of pristine BC declined with the increase of FSHA concentration and the remaining MC-LR in solution gradually increased (Figure 1a). In the presence of 30 mg/L FSHA, 28.82 ± 2.75 μg/L ML-LR remained in solution after the 24 h experiment. This is likely due to the higher aromatic content in FSHA than in MC-LR that competes with MC-LR for the binding sites on BC via π–π interactions.^17^ Furthermore, FSHA (average molar mass 39.098 kDa) can also block BC mesopores to hinder the adsorption of MC-LR (∼1 kDa) by BC.^17,21,39^ Therefore, the presence of FSHA negatively impacted the two major driving forces of MC-LR adsorption by BC (π–π interactions and mesopore filling).^40^ Coating PDDA hydrogel on BC also reduced the mesopore volume (from 0.055 to 0.027 cm^3^/g as shown in Figure S2) but introduced new functional groups to the adsorbent, consequently altered the mechanisms of MC-LR adsorption from pore-filling to chemical attractions, and overcame the drawbacks of BC for MC-LR adsorption in the presence of FSHA.

Next, we examined the effects of co-existing anions (Cl^–^ and SO_4_^2–^) on MC-LR adsorption (Figure 1b,c) because of their potential to alter the electrostatic interactions between the permanent positive charges of PDDA and MC-LR. The results showed that with the increase of Cl^–^ or SO_4_^2–^ concentration from 0 to 200 mg/L, the adsorption efficiency of MC-LR by PDDA–BC remained at >99% during the 24 h equilibrium experiment. A similar result was also observed for pristine BC (Figure 1b,c). This result suggests that the BC fraction of the composite contributes to the adsorption competitivity of MC-LR in the presence of anions. Moreover, electrostatic interactions are not the sole mechanisms that determine the selective adsorption of MC-LR.

The effects of pH (ranging from 2.1 to 11.7) on MC-LR captivation by PDDA–BC in the presence of 30 mg/L of FSHA, SRHA, or SRFA are shown in Figure 1d−f. PDDA–BC maintained a high adsorption efficiency in the presence of FSHA when pH value was below ∼7.2 (Figure 1d). With the increase of pH from 7.2 to 11.7, the adsorption rate significantly declined. A similar trend was also observed for SRHA (Figure 1e). The influence of pH on adsorption is attributable to pH-dependent MC-LR and HA speciation, which influenced their electrostatic interactions with PDDA–BC. In the pH range between 2.1 and 11.7, MC-LRH^–^ with an overall charge of −1 was the dominated species because of the acid dissociation of two carboxylic groups (Figure S3). The grafting of N^+^ group made PDDA–BC display a permanent positive charge under both acidic and alkaline conditions,^28^ as indicated by the XPS results (see Section 3.5). Consequently, MC-LR was attracted to PDDA–BC surface by electrostatic attractions under wide pH range. In comparison, HA was mainly present in the form of positively charged or neutral species when pH was below 7.2 (Figure S4); at pH greater than 7.2, amine and phenolic groups of HA were deprotonated to form negatively charged species. The negatively charged HA would compete for the binding sites with MC-LRH^–^ to reduce the adsorption efficiency of MC-LR.

In comparison, the break point pH for SRFA was approximately 6.1, above which MC-LR adsorption would be significantly inhibited by SRFA (Figures 1f and S5) due to the higher carboxylic content and lower phenolic content of FA than that of HA.^41^ Overall, the adsorption selectivity of MC-LR by PDDA–BC was pH-dependent in the presence of NOM surrogates. The selected NOM surrogate concentration (30 mg/L) used in the study was typical for freshwaters,^42,43^ while the MC-LR concentration was also within common reported ranges from 0 to hundreds of μg/L.^4^ The removal of MC-LR in the presence of NOM surrogates gives insights to its behavior in natural waters. In practical applications, the pH of water often decreases below 7 after the coagulation and flocculation stages of drinking water treatment.^17,44^ Therefore, the application of PDDA–BC for MC-LR removal following this process ensures the maintenance of optimal pH conditions.

### 3.2 Ultrafast Adsorption Kinetics of MC-LR by PDDA–BC

**Figure 2:**
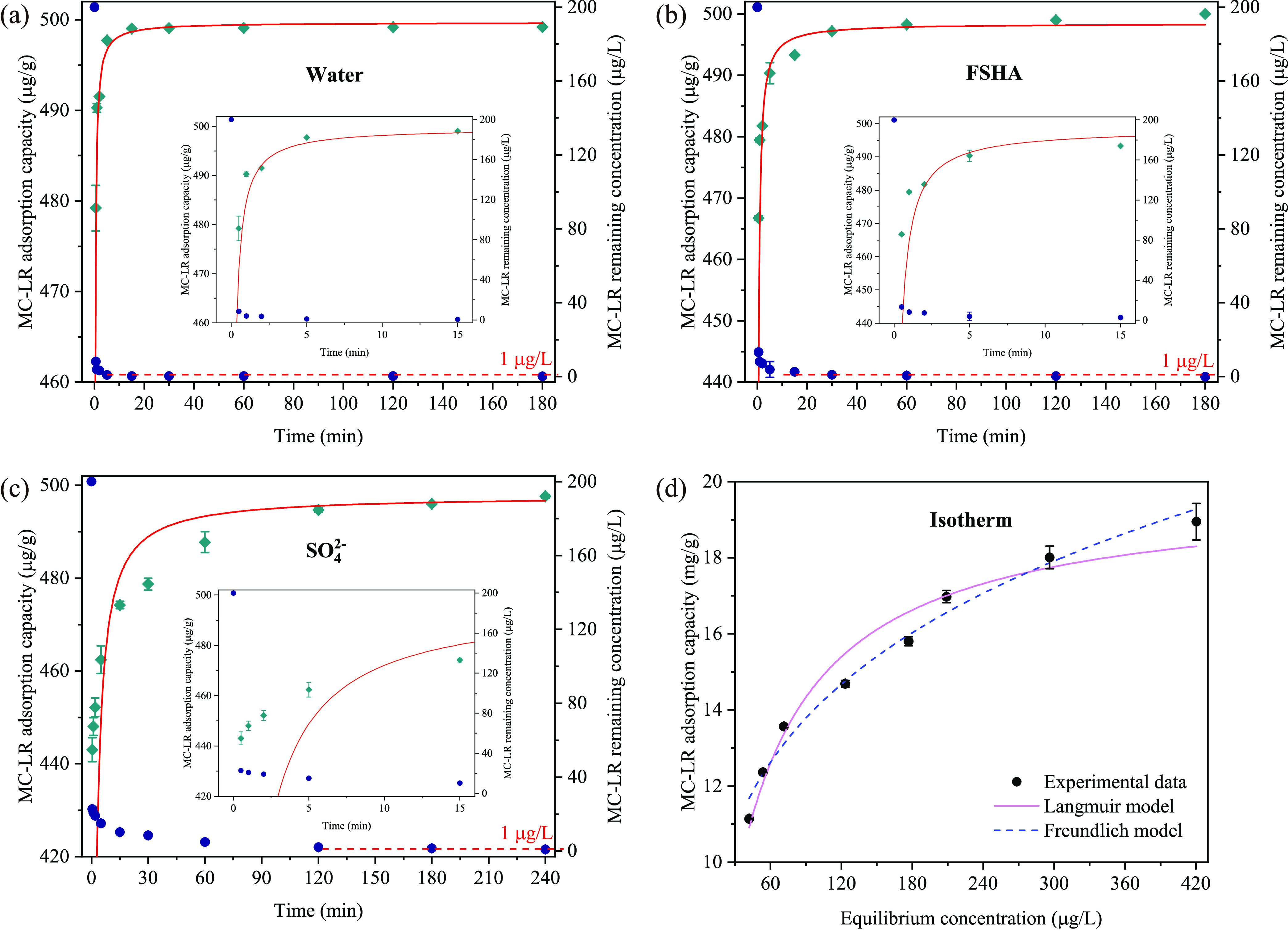
Adsorption kinetics of MC-LR by PDDA–BC in (a) Milli-Q water, (b) 30 mg/L of FSHA, or (c) 200 mg/L of SO_4_^2–^ solution. The green diamonds indicate adsorption capacity (left axis, μg/g) and blue dots show MC-LR remaining in solution (right axis, μg/L). The red line represents the pseudo-second-order kinetics simulation. The inserts in (a–c) are magnified images for MC-LR adsorption kinetics in the first 15 min, and (d) MC-LR adsorption isotherm by PDDA–BC in Milli-Q water fitted by two models.

Adsorption kinetics of MC-LR in Milli-Q water and in the presence of 30 mg/L of FSHA or 200 mg/L of SO_4_^2–^ are shown in Figure 2a–c. An extremely fast kinetics of MC-LR was achieved by PDDA–BC adsorption in Milli-Q water, 98% of MC-LR was captured within 1 min. In the presence of FSHA and SO_4_^2–^, the time required for 98% of MC-LR captivation increased to approximately 5 and 60 min, respectively, suggesting a significant effect of NOM and anions on MC-LR adsorption kinetics. Notably, the effect of co-existing SO_4_^2–^ on MC-LR adsorption kinetics was apparently greater than that of FSHA. It implies that the ultrafast kinetics may result from the increase of positive charge density on the composite, which drives a rapid diffusion of MC-LR to the surface of composite via electrostatic attraction. Furthermore, the remaining MC-LR in solution was reduced to below 1 μg/L (0.91 ± 0.04 μg/L) within 5 min in Milli-Q water, 60 and 240 min in water spiked with FSHA and SO_4_^2–^, respectively. Overall, PDDA–BC shows a great potential to rapidly capture MC-LR and reduce the concentration to below WHO guideline in drinking water treatment. The experimental results were described by the pseudo-second-order model with *R*^2^ values of 0.9564, 0.9485, and 0.8977, respectively. The adsorption rate constants were calculated as 0.0466, 0.01692, and 0.00368 g μg^–1^ min^–1^, respectively (Table S1). It was noted that the model simulation of MC-LR adsorption below 15 min in the presence of SO_4_^2–^ was relatively poor, attributable to the strong effects of SO_4_^2–^ on the diffusion of MC-LR to the surface of PDDA–BC.

Figure 2d shows the isotherm data of MC-LR adsorption in Milli-Q water. The adsorption process was fitted to both Langmuir and Freundlich models with *R*^2^ values of 0.964 and 0.984, respectively (Table S2). The maximum MC-LR uptake value was estimated as 19.79 mg/g by Langmuir model. Apparently, the *R*^2^ value of Freundlich was slightly higher than that of Langmuir, implying a multilayer adsorption on a heterogeneous surface.^45^ This result agrees with previous reports that Freundlich model was well-fitted with the data of MC-LR adsorption on carbon or polymer materials.^21,46−48^ A few others found a better fitting of MC-LR adsorption by Langmuir model.^16,49^ The heterogeneous adsorption in this study may result from the synergistic adsorption mechanisms of electrostatic interactions, π–π stacking, cation−π bonding, and hydrogen bonding for MC-LR adsorption on different binding sites (quaternary ammonium, benzene, and hydroxyl groups, respectively).

### 3.3 Superiority of PDDA–BC for MC-LR Adsorption

**Figure 3:**
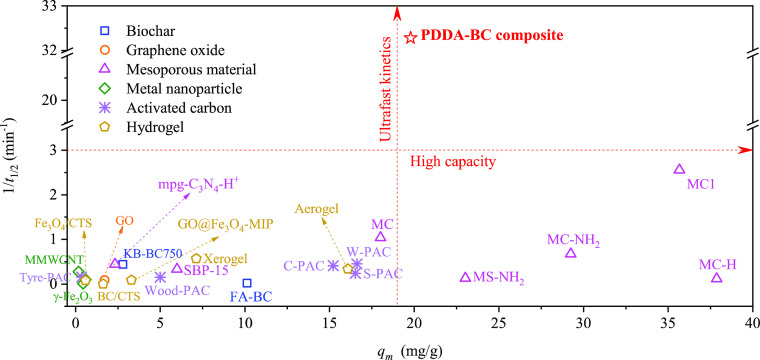
Comparison of MC-LR adsorption by PDDA–BC and by other previously reported adsorbents. Theoretical maximum adsorption capacity (*q*_m_) was calculated from Langmuir isotherm simulation, and inverse of half-life of MC-LR adsorption kinetics (1/*t*_1/2_) was obtained from pseudo-second-order model simulation.

To compare the performance of PDDA–BC with other previously reported adsorbents, 20 published reports about MC-LR adsorption were examined to obtain the MC-LR adsorption kinetics and isotherm data in pure water of 27 different adsorbents (Figure 3 and Table S3). These adsorbents were classified as the various types of BC, graphene oxide, mesoporous material, metal nanoparticle, activated carbon, and hydrogel. The *Kq*_*k*_ values from pseudo-second-order model (the inverse of the half-life of adsorption process)^50^ were used to compare the adsorption rates. The maximum adsorption capacity was calculated to compare the affinity of MC-LR removal between PDDA–BC and other adsorbents.

As shown in Figure 3, the adsorption kinetics of PDDA–BC far exceeded the other adsorbents in pure water. The *Kq*_*k*_ of PDDA–BC was 22.28 min^–1^, which was 8.7 to 10^4^ times higher than that of other adsorbents. That is, PDDA–BC required 8.7 to 10^4^ times shorter time than the surveyed adsorbents to achieve 50% of MC-LR equilibrium adsorption capacity. The PDDA–BC also showed a high adsorption affinity toward MC-LR with maximum adsorption capacity of 19.79 mg/g, which was obviously higher than that of most surveyed adsorbents except mesoporous materials. The literature reports suggest that mesoporous materials that contain 2–50 nm diameter pores are effective to capture MC-LR molecules (1.1 × 1.9 × 1.5 nm) because pore filling plays an important role in the MC-LR removal.^14,51^ However, in the presence of NOM (humic acid or fulvic acid), a significant decline of MC-LR adsorption has been observed because of the blocking of mesopores by competing organic molecules.^17,21^ Therefore, common mesoporous materials have poor adsorption selectivity toward MC-LR in the presence of NOM. In comparison, the PDDA–BC presents a distinctive advantage in the efficient and selective captivation of MC-LR in the presence of NOM or anions as demonstrated earlier. Overall, the PDDA–BC offers exceptional kinetics, capacity, and selectivity for MC-LR adsorption and has a greater potential than the surveyed adsorbents for implementation in engineered water treatment.

### 3.4 PDDA–BC for MC-LR Adsorption in Natural Lake Water

**Figure 4:**
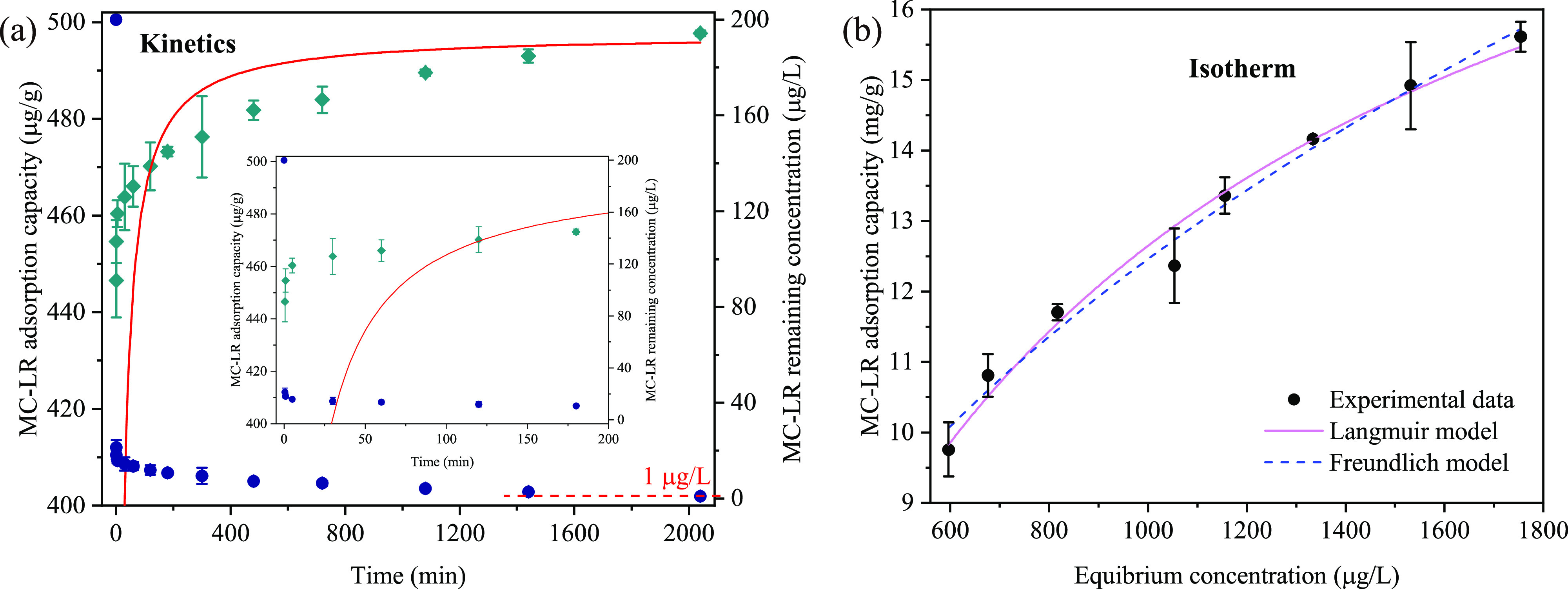
MC-LR adsorption in lake water. (a) PDDA–BC adsorption kinetics of MC-LR in the spiked lake water (200 μg/L). The green diamonds indicate adsorption capacity (left axis, μg/g) and blue dots show MC-LR remaining in lake water (right axis, μg/L). The red line represents the pseudo-second-order kinetics simulation. The insert in (a) is a magnified image for MC-LR adsorption kinetics in the first 200 min. (b) Adsorption isotherm of MC-LR by PDDA–BC in the spiked lake water (4.5–8 mg/L).

The effectiveness of the PDDA–BC for MC-LR removal was further evaluated in the lake water samples. The UV254 absorbance and electrical conductivity of the lake water were 0.096 ± 0.006 cm^–1^ and 1258.5 ± 26.1 μs/cm, respectively, indicating the presence of NOM and ions. MC-LR was not detected in the lake water at the time of sampling. Batch experiments with MC-LR spiked lake water showed that more than 90% of MC-LR (200 μg/L) was rapidly captured by PDDA–BC within 1 min (Figure 4a). MC-LR level dropped below 1 μg/L after 34 h adsorption. In comparison with the adsorption in Milli-Q water or in the presence of FSHA or SO_4_^2–^, the adsorption rate in the lake water evidently decreased. It is attributed to the complex composition of lake water that contains various NOM and inorganic compounds. Considering inorganic ions can interact with NOM, co-existing of these compounds in natural water is likely to have a complex effect on MC-LR adsorption^52^ that requires future investigations. Nevertheless, the results of lake water study showed that the MC-LR adsorption kinetics was described by the pseudo-second-order model (Table S1). The adsorption isotherm data were fitted to Langmuir and Freundlich models (Figure 4b and Table S2). Consistent with the results in synthetic water matrix, Freundlich provided a better description for the MC-LR adsorption in lake water. The maximum adsorption capacity of MC-LR obtained from Langmuir model was 21.99 mg/g, which was similar to the modeling results in Milli-Q water. This result indicates that the majority of the binding sites on PDDA–BC favor MC-LR in the presence of high concentration of NOM and inorganic compounds and can selectively capture MC-LR. The PDDA–BC maintained a high adsorption capacity toward MC-LR, which demonstrates the potential for the captivation of MC-LR in natural water.

### 3.5 Mechanistic Insights into MC-LR Adsorption by PDDA–BC

**Figure 5:**
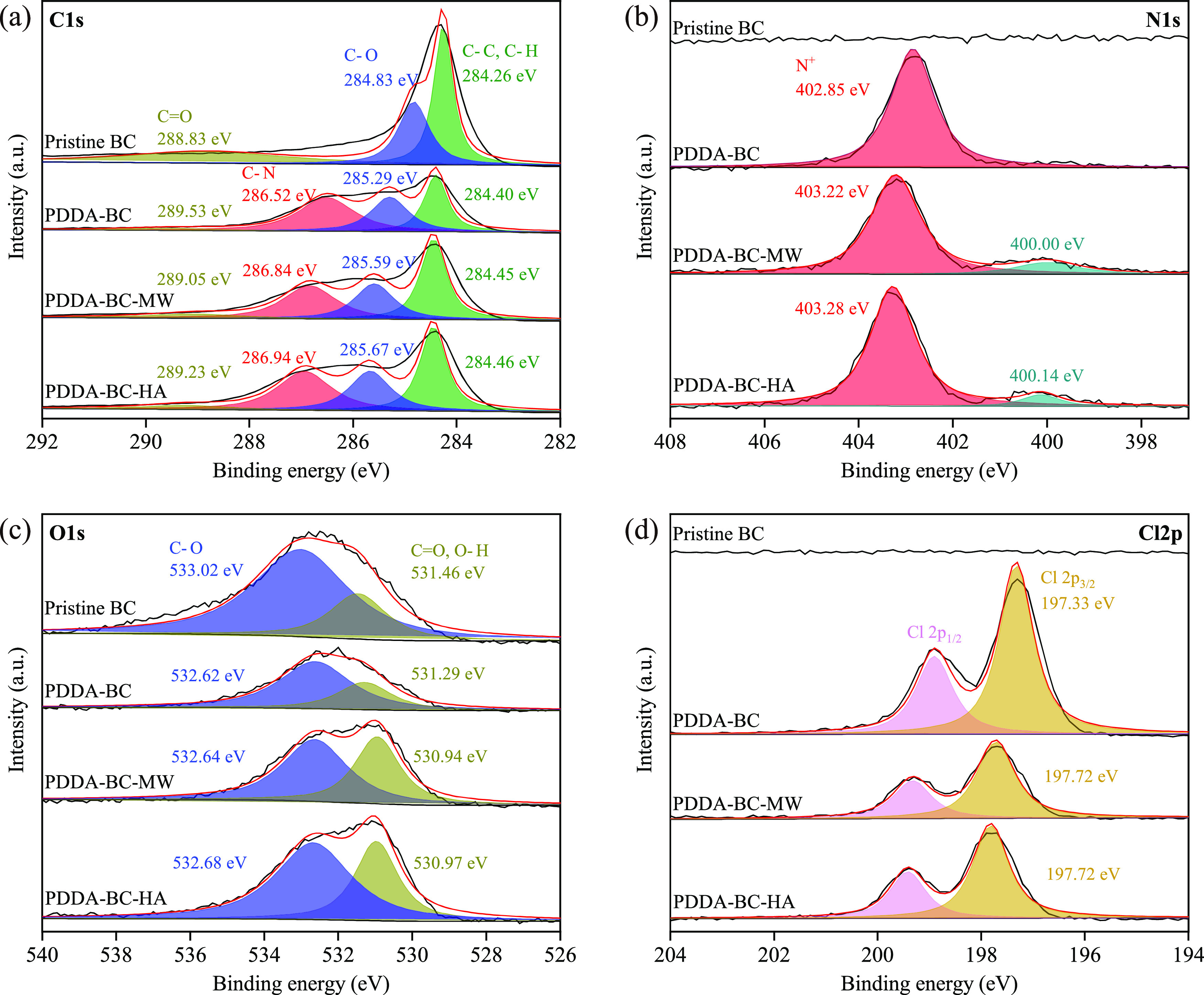
XPS spectra of (a) C 1s, (b) N 1s, (c) O 1s, and (d) Cl 2p for pristine BC and PDDA–BC before and after MC-LR adsorption. PDDA–BC–MW represents the PDDA–BC after adsorbing MC-LR in Milli-Q water; and PDDA–BC–HA represents the PDDA–BC after adsorbing MC-LR in 30 mg/L of FSHA solution.

XPS analysis of BC and PDDA–BC revealed the plausible mechanisms of MC-LR removal (Figures 5 and S5). For C 1s spectrum, pristine BC showed three peaks at 288.83, 284.83, and 284.26 eV, corresponding to C=O, C–O, and C–C/C–H, respectively (Figure 5a).^53^ Besides these three peaks, a new peak assigned to C–N at 286.52 eV appeared in the C 1s spectrum of PDDA–BC. Accordingly, the peak of N^+^ group at 402.85 eV^54^ was clearly observed in the N 1s spectrum of PDDA–BC, whereas not in pristine BC (Figure 5b). These results confirmed that PDDA hydrogel was successfully coated on the BC to form a composite. After MC-LR adsorption in Milli-Q water, a new peak at 400.00 was observed in the N 1s spectrum, which can be assigned to a secondary amine group.^55^ Similarly, experiment in FSHA solution resulted in a 400.14 eV peak. It is ascribed to the high content of secondary amine group in MC-LR and provides a direct evidence that MC-LR was adsorbed to PDDA–BC. This interpretation is further confirmed by the result of XPS O 1s. The O 1s spectrum can be deconvoluted into two peaks for C–O and C=O/O–H, respectively.^56,57^ The fractions of the C=O/O–H groups increased from 30.50 to 42.73% after adsorbing MC-LR in the Milli-Q water and to 37.06% in the experiment with FSHA solution (Figure 5c). Meanwhile, the peaks of the C=O/O–H groups significantly shifted to a lower binding energy from 531.29 to 530.94 and 530.97 eV, respectively. These are likely caused by the abundant C=O groups in MC-LR. Importantly, the peaks of N^+^ in N 1s spectrum significantly shifted to a higher binding energy from 402.85 to 403.22 and 403.28 eV, respectively (Figure 5d). This increase may be from the bonding of N^+^ with MC-LR through replacing Cl^–^, which increases the electron cloud density around nitrogen atoms. This reasoning is supported by the marked decrease of Cl 2p peak intensity after adsorbing MC-LR (Figure 5d). Therefore, the N^+^ groups of PDDA–BC acted as an important binding site for the captivation of MC-LR in adsorption.

Analysis of XPS spectra together with MC-LR molecular structure revealed that the N^+^ groups of PDDA–BC can interact with the deprotonated carboxylic groups of d-Glu and d-Masp in the MC-LR structure (Figure S7) through electrostatic interaction.^58^ Moreover, cation−π bonding may occur between the N^+^ groups of PDDA–BC and the benzene ring of MC-LR during the adsorption process.^59^ Given the benzene ring in the MC-LR structure, the aromatic structure of the PDDA–BC may provide active sites for MC-LR adsorption through π–π stacking.^60^ On the basis of XPS analysis, the PDDA–BC contains abundant hydroxyl groups, and they may serve as the interaction sites of hydrogen bonding for MC-LR adsorption with the carbonyl, hydroxyl, or amine groups in the MC-LR structure.^58,61^ Overall, XPS results support that electrostatic interactions, cation−π bonding, π–π stacking, and hydrogen bonding were involved in the adsorption of MC-LR by PDDA–BC.

**Figure 6:**
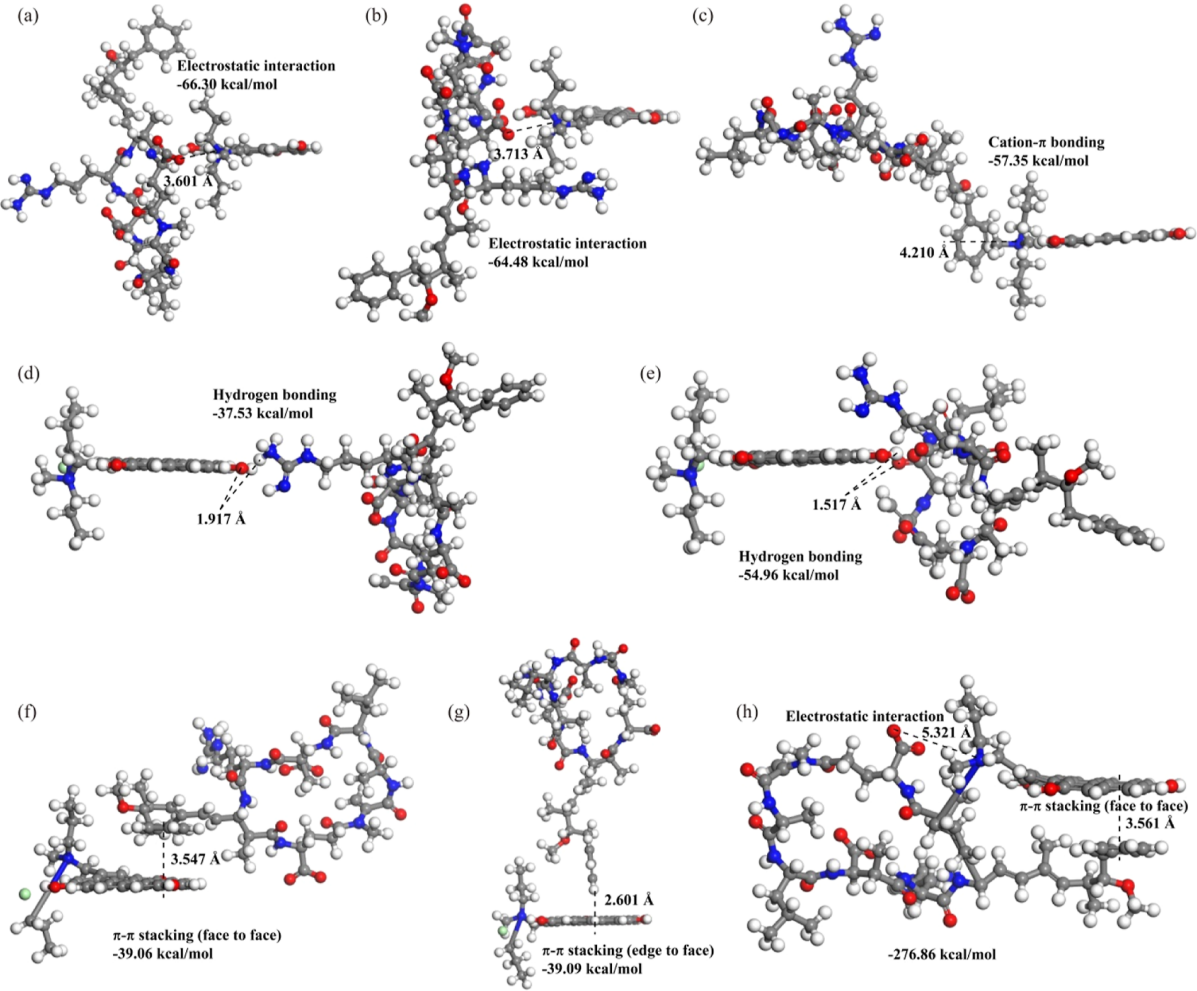
Optimized geometries for the interactions of MC-LR with PDDA–BC. (a) Electrostatic interaction with d-Glu carboxylic group of MC-LR as binding site, (b) electrostatic interaction with d-Masp carboxylic group of MC-LR as binding site, (c) cation−π bonding, (d) hydrogen bonding with H–O···H–N, (e) hydrogen bonding with O–H···O=C–O^–^, (f) π–π stacking for face to face interaction, (g) π–π stacking for edge to face interaction, and (h) electrostatic interaction and π–π stacking for face to face position.

DFT simulation was then further performed to confirm the XPS observations. The binding energies of d-Glu and d-Masp carboxyl with the N^+^ groups of PDDA–BC were calculated as −66.30 and −64.48 kcal/mol, respectively (Figure 6a,b). In comparison, the energies of other interactions ranging from −57.35 to −37.53 kcal/mol were significantly higher than that of electrostatic interactions, following the order of electrostatic interaction < cation−π bonding < hydrogen bonding < π–π stacking (Figure 6c–g). This result is consistent with the observation in XPS analysis that the N^+^ groups played important roles in MC-LR removal. The introduction of N^+^ groups may be the essential reason for the ultrafast kinetics of MC-LR adsorption.

To evaluate the adsorption selectivity of MC-LR, we further calculated the adsorption energy between Cl^–^ anion and PDDA–BC (Figure S8) and the value was −109.25 kcal/mol. It means that Cl^–^ has a stronger affinity than MC-LR to be adsorbed to the PDDA–BC, which conflicts with the experimental observation of fast kinetics of MC-LR in the presence of Cl^–^ and the decrease of XPS Cl 2p peak intensity after adsorption. Furthermore, the single electrostatic interaction, hydrogen bonding, or π–π stacking for the SRFA was also stronger than that for MC-LR adsorption onto the PDDA–BC when the pH was about 6 (Figures S8 and S9). However, according to the experiments of MC-LR adsorption in the presence of SRFA, PDDA–BC showed a high selectivity at pH = 6.02. These results indicate that any single binding mechanism of anions or NOM was not strong enough to outcompete MC-LR for the binding sites on PDDA–BC. Multiple interactions acted simultaneously for PDDA–BC adsorption of MC-LR, which induce a stronger binding between the MC-LR and the composite. Optimizing the configuration of MC-LR adsorption onto the PDDA–BC revealed that the energy was lowest (−276.86 kcal/mol) when electrostatic interaction (d-Glu carboxyl) and π–π stacking simultaneously occurred during the MC-LR adsorption (Figure 6h), which was consistent with the previous reports about organic pollutant removal.^26,60^ The synergetic effect of electrostatic interaction and π–π stacking is the key to the selective and efficient adsorption of MC-LR.

### 3.6 Coupling Adsorption–Biodegradation for Removal of MC-LR

**Figure 7:**
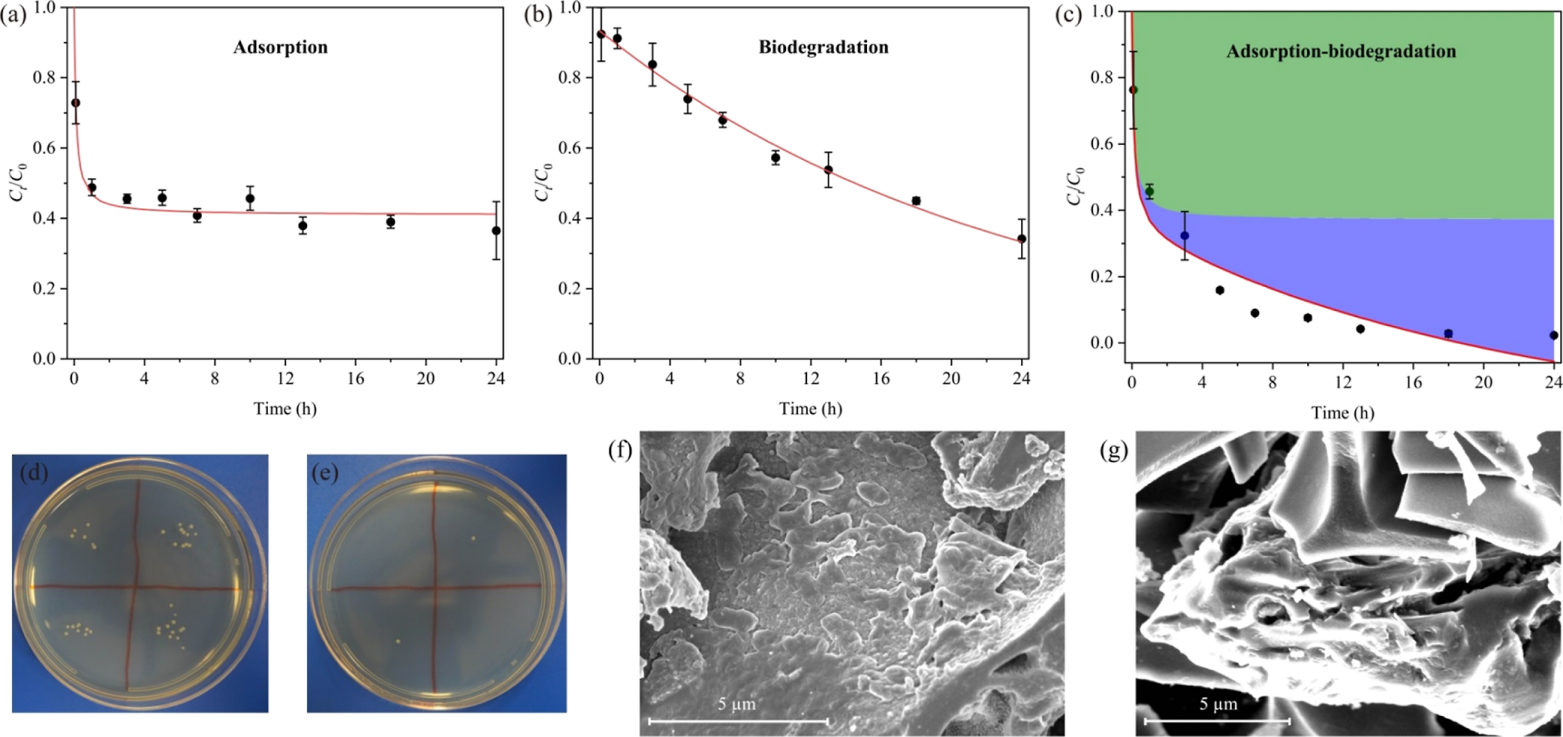
Removal kinetics of MC-LR and modeling results by (a) PDDA–BC adsorption, (b) *Sphingopyxis* sp. m6 biodegradation, and (c) coupled PDDA–BC adsorption and *Sphingopyxis* sp. m6 biodegradation. The black dots and red lines represent the experimental and modeling results, respectively. The green and blue shade in (c) represent the adsorption and coupled adsorption–biodegradation contribution to MC-LR removal, respectively. A GLM was used to calculate the contribution of adsorption and biodegradation to MC-LR removal and the equation is shown in Supporting Information. Plate cultivation results of *Sphingopyxis* sp. m6 in PDDA–BC mixture (d) and unattached bacteria in the filtrate (e). SEM images of PDDA–BC after co-culturing with *Sphingopyxis* sp. m6 (f) and pure PDDA–BC (g).

Figure 7a shows the adsorption kinetics of MC-LR with initial concentration of 7 mg/L in M9 media. The data were fitted to pseudo-second-order model with the adsorption capacity and rate constant of 10.33 mg/g and 0.90444 g mg^–1^ h^–1^, respectively (Table S4). These parameters are smaller than those obtained in the Milli-Q water or lake water due to the presence of high salts in M9 media that affected the electrostatic interactions between PDDA–BC and MC-LR. In comparison, *Sphingopyxis* sp. m6 grown in M9 media was capable of degrading MC-LR to a lower concentration, but at slower kinetics (Figure 7b) that was well described by first-order model (Table S4). Interestingly, the MC-LR removal efficiency in the PDDA–BC–bacteria mixture was significantly higher than that by adsorption alone or biodegradation alone. This is likely due to the coupled interaction of adsorption and biodegradation. We expressed coupled interaction as a GLM to estimate their contribution to MC-LR removal.^38^ As shown in Figure 7c and Table S4, GLM was sufficient to describe the adsorption and biodegradation removal of MC-LR. The results showed that adsorption alone contributed 49.33% of MC-LR removal; adsorption–biodegradation contributed 50.67%.

According to bacterial plate counts, 94.7% of the *Sphingopyxis* sp. m6 were attached to the surface of PDDA–BC and only 5.3% survived as the free-living form (Figure 7d,e). Bacterial attachments on PDDA–BC composite were also revealed by the SEM images, which showed smooth layers of biofilm in comparison to the sharp rigid structure of PDDA–BC without bacterial colonization (Figure 7f,g). It has been confirmed that BC can act as an “electron shuttle” to directly mediate the electron transfer between microbes and organics/minerals.^62,63^ The degradation of MC-LR by attached *Sphingopyxis* sp. m6 on PDDA–BC may attribute to the electron transfers by the BC. Therefore, the results suggest that MC-LR was first selectively and rapidly captured by the PDDA–BC and then was degraded by attached bacteria to nontoxic products.^9^ The adsorption–biodegradation process potentially overcomes the drawbacks of either pure adsorption or biodegradation alone and offers a new sustainable approach for MC-LR removal in practical use.

### 3.7 Environmental Implications

The PDDA–BC composite presented ultrafast adsorption kinetics and high affinity toward MC-LR, rapidly capturing MC-LR and reducing the concentration in water to below 1 μg/L even in the presence of competing NOM and anions. The PDDA–BC outperformed previously reported adsorbents with respect to adsorption kinetics and capacity. The adsorption selectivity of MC-LR in the presence of NOM was pH-dependent. Thus, PDDA–BC should be used following coagulation–flocculation during drinking water treatment to achieve a selective captivation of MC-LR in practical applications. Importantly, although the adsorption kinetics in natural lake water was slower than that in synthetic water, results showed that PDDA–BC was sufficiently effective capturing MC-LR in the natural lake water.

The adsorption mechanisms of MC-LR by the PDDA–BC were theoretically and experimentally confirmed, which benefit the redesign and optimization of the biomaterial. The synergetic effect of electrostatic interaction and π–π stacking resulted in the strong affinity of PDDA–BC toward MC-LR, which benefited the adsorption of MC-LR in natural water matrices. Furthermore, the coupling of PDDA–BC adsorption with MC-degrading bacteria was successfully demonstrated. The coupled adsorption–biodegradation technology could overcome the drawbacks of single adsorption or biodegradation. Immobilization of MC-LR on the PDDA–BC composite provides the opportunity for the complete biodegradation of the toxin by bacteria at slower kinetics. Breakdown of the MC-LR by biodegradation releases active binding sites on the composite for re-adsorption of new molecules. Therefore, adsorption–biodegradation could be a sustainable strategy in designing engineered biofiltration for MC-LR elimination. Overall, the study outcomes imply that PDDA–BC has practical potentials to capture MC-LR in environmental water matrices. The understandings of the adsorption performance of the biomaterial provide the adsorption parameters (such as adsorption rate, maximum adsorption capacity, and selectivity) for the design of column experiments in the next phase of our investigation (such as the hydraulic retention time and the breakthrough point). We will synthesize material-bacteria beads as the media of column experiments and investigate the removal of MC-LR in the biofilter. We will evaluate the performance of MC-LR removal in a continuously flow through system and understand the shock resistance and stability of the system in the synthetic water (containing both NOM surrogates and ions) or natural water. The operation of the biofiltration systems is needed to evaluate its practical use in engineering treatment of MC-LR polluted water.

### 3.8 Conclusions

The PDDA–BC showed a selective and ultrafast adsorption toward MC-LR. The effects of NOM (FSHA, SRHA, and SRFA) on the MC-LR adsorption were very slight when solution pH was below 7. The removal of MC-LR (200 μg/L) was >99% during a 24 h equilibrium experiment in the presence of Cl^–^ or SO_4_^2–^ (0–200 mg/L). The 98% of MC-LR was removed within 1 min in Milli-Q water, within 5 min in the presence of 30 mg/L of FSHA and 60 min in solution of 200 mg/L of SO_4_^2–^. The adsorption isotherm was better described by Freundlich model than by Langmuir model. The performance of PDDA–BC was largely maintained in natural lake waters that were spiked with MC-LR, indicating its good engineering practicability. The DFT calculations revealed that the synergetic effect of electrostatic interaction and π–π stacking was the key to the selective and efficient removal of MC-LR. The experiment results confirmed that the coupling of PDDA–BC with MC-degrading bacteria had a better removal of MC-LR than single adsorption or biodegradation. The PDDA–BC that can selectively and efficiently capture MC-LR will facilitate biodegradation during biofiltration.

## Supporting Information Available

Sources and description of materials used; adsorption kinetics, isotherm and biodegradation modeling and results; MC-LR adsorption abilities of different adsorbents in the literature; geometry optimization of PDDA–BC model; pore size distribution of PDDA–BC and BC; speciation of MC-LR, HA, and FA in solution at different pH conditions; wide-scan XPS spectra of pristine BC and PDDA–BC; DFT models of PDDA–BC, MC-LR, and SRFA; DFT calculation results for the interactions between Cl^–^ and PDDA–BC; and DFT calculation results for the interactions between SRFA and PDDA–BC (PDF)
