# Supplementary material for: Immobilization of Microcystin by the Hydrogel–Biochar Composite to Enhance Biodegradation during Drinking Water Treatment
Source: ACS ES T Water. 2023 Aug 29;3(9):3044–56. doi: 10.1021/acsestwater.3c00240 (PMC10496130; doi:10.1021/acsestwater.3c00240)
Supplement: Supplementary file 1 — ew3c00240_si_001.pdf [file ew3c00240_si_001.pdf]

Immobilization of Microcystin by Hydrogel-Biochar Composite to Enhance Biodegradation during Drinking Water Treatment

Lixun Zhang, Shengyin Tang, and Sunny C. Jiang*

Department of Civil and Environmental Engineering, University of California, Irvine, 92697, United States

* Corresponding authors: sjiang@uci.edu.

Supporting Information

1.1 Supplemental materials information used in the study

Dry MC-LR (500 μg powder) was purchased from MilliporeSigma™ (Burlington, MA, United States), and was dissolved in methanol and then diluted using Milli-Q water to prepare stock solutions. Nodularin solution (10 μg/mL in methanol) was obtained from Honeywell Fluka™ (Mexico City, Mexico) and applied as an internal standard of MC-LR analysis by UPLC-MS/MS. Humic acids represent a broad range of structurally complex compounds containing aliphatic, aromatic, and hydrophilic functional groups. The humic acids from different companies may have different characteristics. Therefore, Suwannee river humic acid (SRHA) and Fisher Scientific humic acid (FSHA) were purchased from the International Humic Substances Society and Fisher Scientific respectively to fully investigate the effects of humic acids on MC-LR adsorption. Moreover, flulvic acids represent smaller and less hydrophobic NOM compounds than humic acids, thus, Suwannee river fulvic acid (SRFA) was also purchased from the International Humic Substances Society as a representative of NOM. Pristine BC was supplied by Lewis Bamboo Inc. (Alabama, United States). Poly(diallyldimethylammonium chloride) (PDDA, 20 wt% in water, 1.04 g/mL and 600−900 cP at 25°C) was acquired from Sigma-Aldrich (St. Louis, MO, United States). All experiment solutions were prepared using deionized water (18.2 MΩ·cm) (Milli-Q, Millipore).

1.2 Adsorption kinetics modeling

Adsorption kinetics of MC-LR by PDDA-BC were simulated using pseudo-second-order (Eq. S1) model.

$$\frac{t}{q_t} = \frac{1}{K_1 q_k^2} + \frac{t}{q_k} \qquad \text{(S1)}$$

where, q_k (μg/g) is the adsorption capacity of the adsorbent after equilibrium; q_t (μg/g) is the adsorption capacity of the adsorbent at time t (min); and K_1 (g·μg^{-1}·min^{-1}) is the pseudo-second-order rate constant.

1.3 Adsorption isotherm modeling

Adsorption isotherms of MC-LR by PDDA-BC were simulated using Langmuir (Eq. S2) and Freundlich (Eq. S3) models.

$$q_i = \frac{q_m \times b \times C_i}{1 + b \times C_i} \tag{S2}$$

$$q_i = K_f \times C_i^{(1/n)} \tag{S3}$$

where, q_i (mg/g) is the adsorption capacity of the adsorbent; C_i (mg/L) is the equilibrium concentration; b (L/mg) is the Langmuir bonding term; K_f (L^{n}·mg$^{(1-n)}$·g^{-1}) is the Freundlich affinity coefficient; q_m (mg/g) is the Langmuir maximum adsorption capacity; and n is the Freundlich linearity constant.

1.4 Biodegradation modeling

Biodegradation of MC-LR by *Sphingopyxis* sp. m6 was simulated using first-order kinetics model (Eq. S4).

$$C_t = C_0 \times e^{-K_2 t} \tag{S4}$$

where, C_i (mg/L) is the equilibrium concentration; C_0 (mg/L) is the initial concentration; and K_2 (h^{-1}) is the rate constant.

1.5 Generalized linear modeling

$$E(Y) = g^{-1}(a \times x + b \times y + c \times x \times y) \tag{S5}$$

where, $E(Y)$ is the MC-LR removal efficiency; g is the link function that specifies how the MC-LR removal relates to the two mechanisms, and identity link was used herein; x

represents the MC-LR adsorption removal that is fitted to pseudo-second-order kinetic model; y represents the MC-LR biodegradation that is described by first-order kinetic model; $x \times y$ represents the interaction of adsorption and biodegradation for MC-LR removal; a, b, and c are the constants that are related to the contribution of each part to MC-LR removal.

2. Supplemental Tables

Table S1 Parameters of pseudo-second-order kinetics models for MC-LR adsorption.

Parameters	Treatments			
	Milli-Q water	30 mg/L of FSHA solution	200 mg/L of SO_4^{2-} solution	Lake water
R^2	0.9564	0.9485	0.8977	0.8914
q_k (μg/g)	499.70	498.45	496.96	497.65
K (g·μg^{-1}·min^{-1})	0.06460	0.02765	0.00246	1.35813E-4

Table S2 Parameters of Langmuir and Freundlich isotherm models for MC-LR adsorption.

Isotherm model	Parameters	Treatments	
		Milli-Q water	Lake water
Langmuir	R^2	0.9640	0.9842
	q_m (mg/g)	19.79	21.99
	b (L/mg)	0.02916	0.00135
Freundlich	R^2	0.9840	0.9870
	K_f	5.17	0.71
	n	4.59	2.41

Table S3 Comparison of Langmuir maximum adsorption capacity (q_m) and inverse of adsorption kinetics half-life (K_1q_k) of different adsorbents for MC-LR adsorption. All kinetics experiments were conducted in MC-LR spiked pure water or Milli-Q water.

Adsorbents	Type	q_m (mg/g)	K_1q_k (min^{-1})	References
FA-BC	Biochar	10.12	0.021	1
KB-BC750	Biochar	2.78	0.443	2
GO	Graphene oxide	1.70	0.092	3
MC1	Mesoporous material	35.67	2.556	4
MC-H	Mesoporous material	37.87	0.117	5
MC-NH_2	Mesoporous material	29.25	0.675	5
mpg-C_3N_4-H^+	Mesoporous material	2.32	0.444	6
MC	Mesoporous material	18.01	1.038	7
MS-NH_2	Mesoporous material	23.03	0.128	5
SBP-15	Mesoporous material	5.99	0.329	8
α-Fe_2O_3	Metal nanoparticle	0.59	0.104	9
γ-Fe_2O_3	Metal nanoparticle	0.44	0.009	10
MMWCNT	Metal nanoparticle	0.17	0.277	11
Tyre-PAC	Powered activated carbon	0.36	0.156	12
Wood-PAC	Powered activated carbon	5.00	0.153	13
S-PAC	Powered activated carbon	16.53	0.240	7
C-PAC	Powered activated carbon	15.22	0.409	7
W-PAC	Powered activated carbon	16.62	0.454	7
Fe_3O_4/CTS	Hydrogel	0.59	0.085	14
GO@Fe_3O_4-MIP	Hydrogel	3.30	0.090	15
Xerogel	Hydrogel	7.13	0.572	16
Aerogel	Hydrogel	16.08	0.338	16
BC/CTS	Hydrogel	1.63	0.002	17
**PDDA-BC**		**19.79**	**32.281**	**This study**

Table S4 Modeling results of adsorption, biodegradation, and coupled adsorption-biodegradation of MC-LR in M9 media.

Models	Parameters	
Pseudo-second-order model for adsorption	R^2	0.9011
	q_k (mg/g)	10.33
	K_1 ($g{\cdot}mg^{-1}{\cdot}h^{-1}$)	0.90444
First-order model for biodegradation	R^2	0.9912
	C_0 (mg/L)	6.53
	K_2 (h^{-1})	0.04297
Generalized linear modeling for adsorption-biodegradation	R^2	0.9041
	a	1.066
	b	1.07×10^{-14}
	c	1.095

3. Supplementary Figures

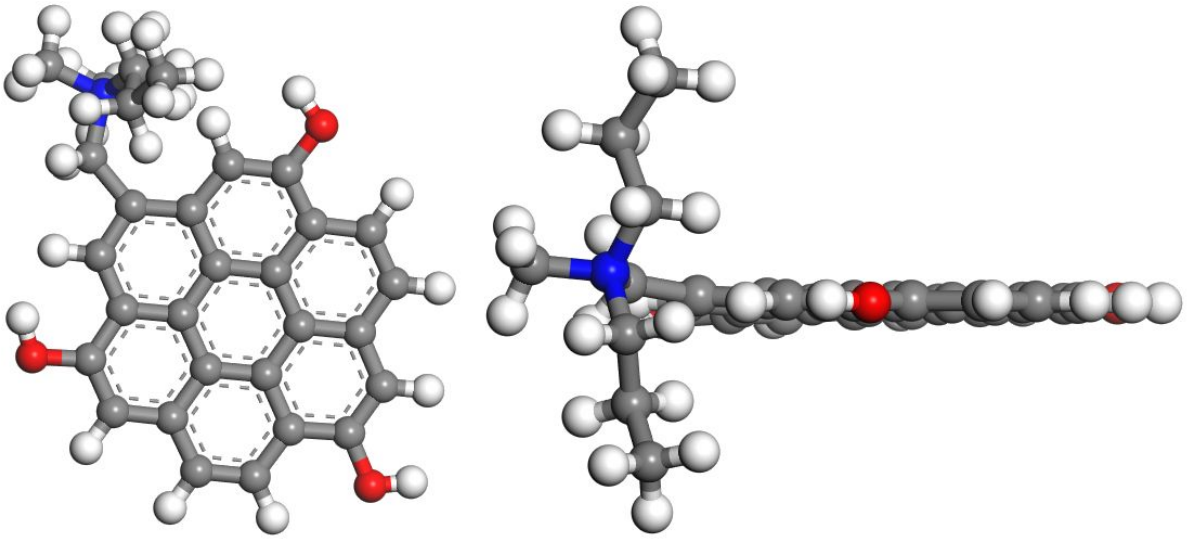

Figure S1 Geometry optimization of PDDA-BC model using Dmol3 package.

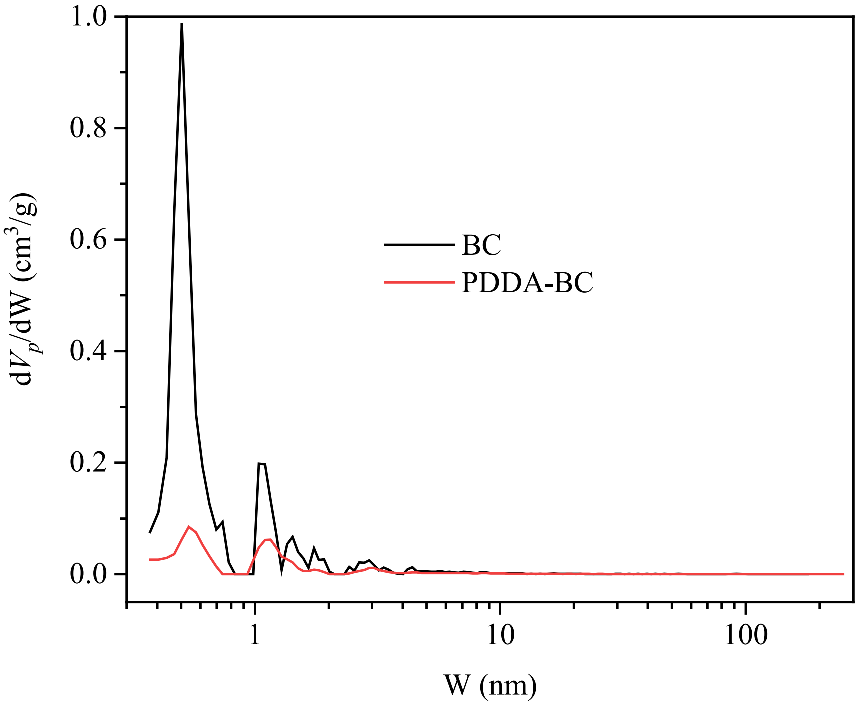

Figure S2 Pore size distributions of BC and PDDA-BC calculated using NLDFT/GCMC model (Vp is the pore volume and W is the pore size).

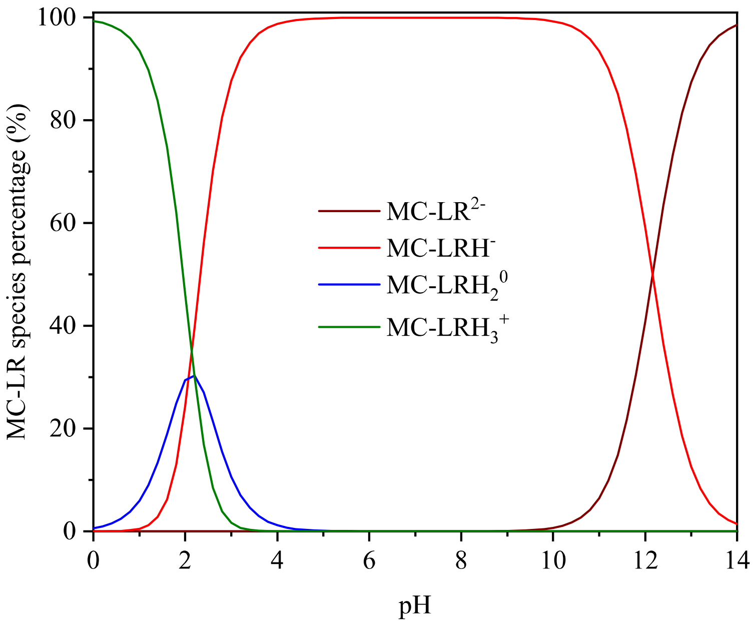

Figure S3 Speciation distribution of MC-LR in solution at different pH conditions. The pKa values of MC-LR are 2.09, 2.19, and 12.48.[18]

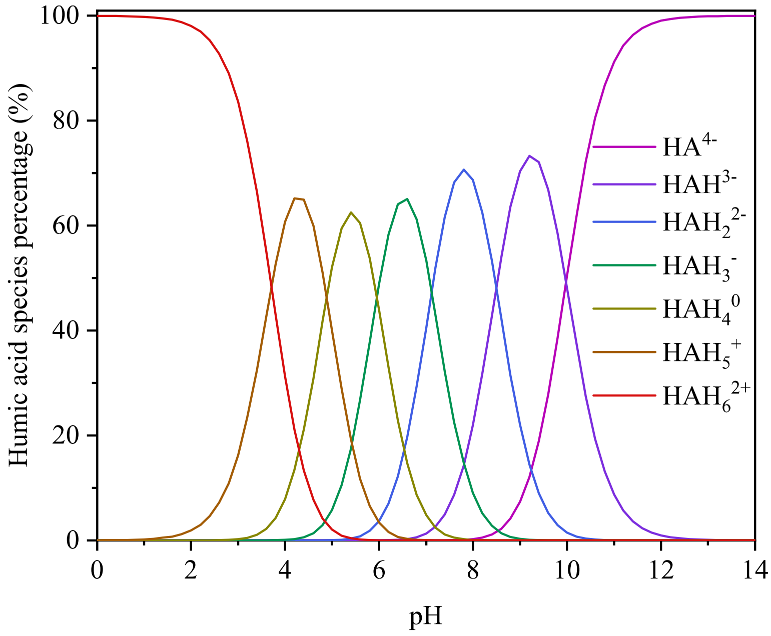

Figure S4 Speciation distribution of humic acid in solution at different pH conditions. The pKa values of humic acid are 3.39, 4.78, 6.06, 7.44, 9.03, and 10.73.[19]

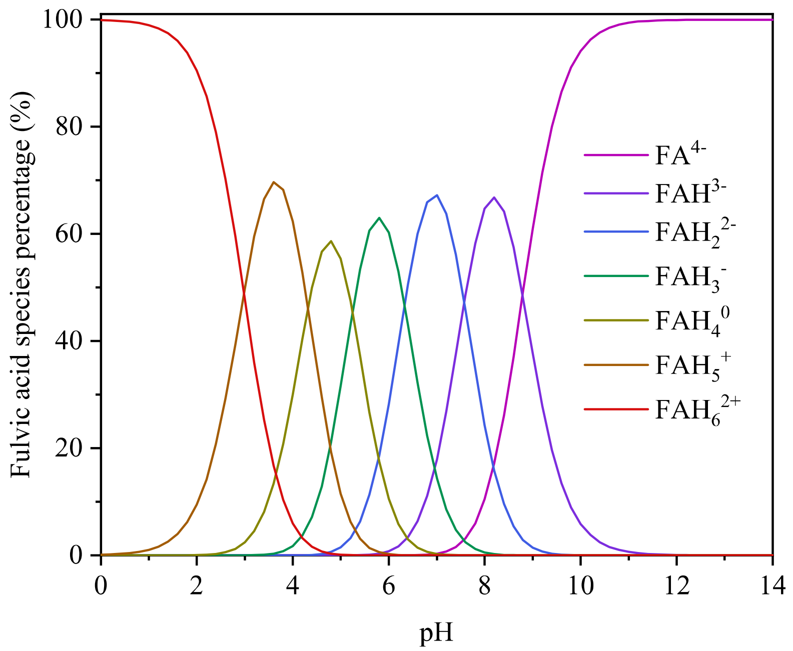

Figure S5 Speciation distribution of fulvic acid in solution at different pH conditions. The pKa values of fulvic acid are 2.66, 4.21, 5.35, 6.65, 8.11, and 9.54.[19]

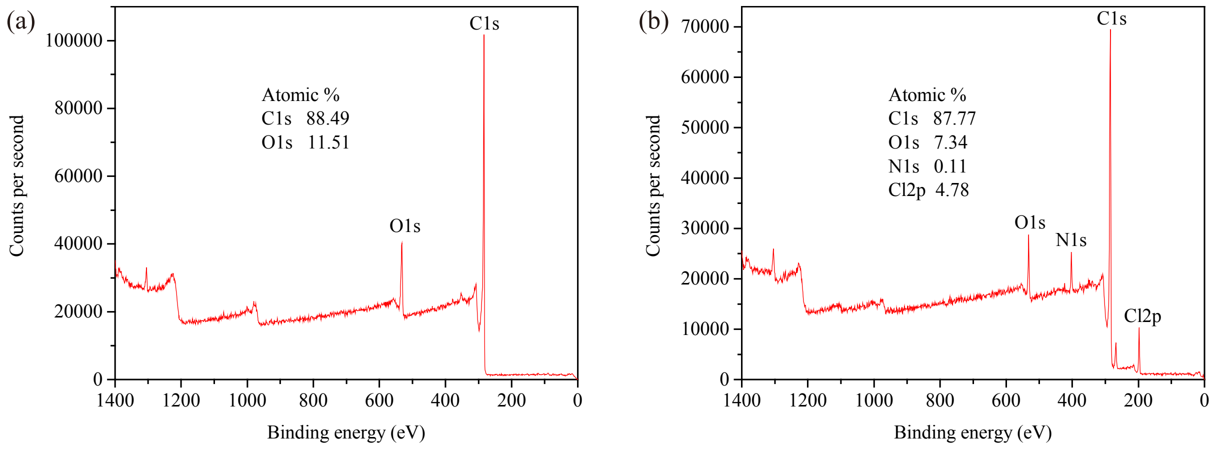

Figure S6 Wide-scan XPS spectra of pristine biochar (a) and PDDA-BC (b).

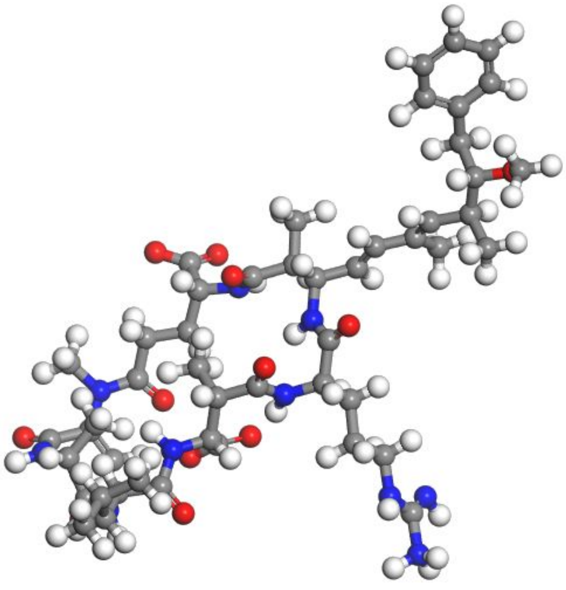

Figure S7 Geometry optimization of MC-LR using Dmol3 package.

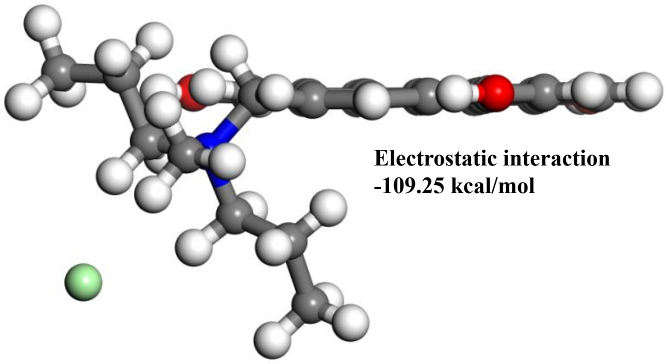

Figure S8 Optimal configurations of electrostatic interaction between Cl^- and PDDA-BC via DFT calculations.

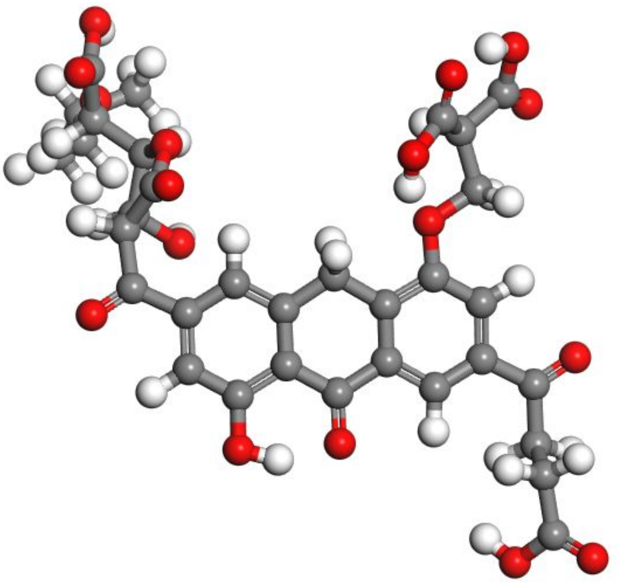

Figure S9 Geometry optimization of Suwannee river fulvic acid using Dmol3 package.

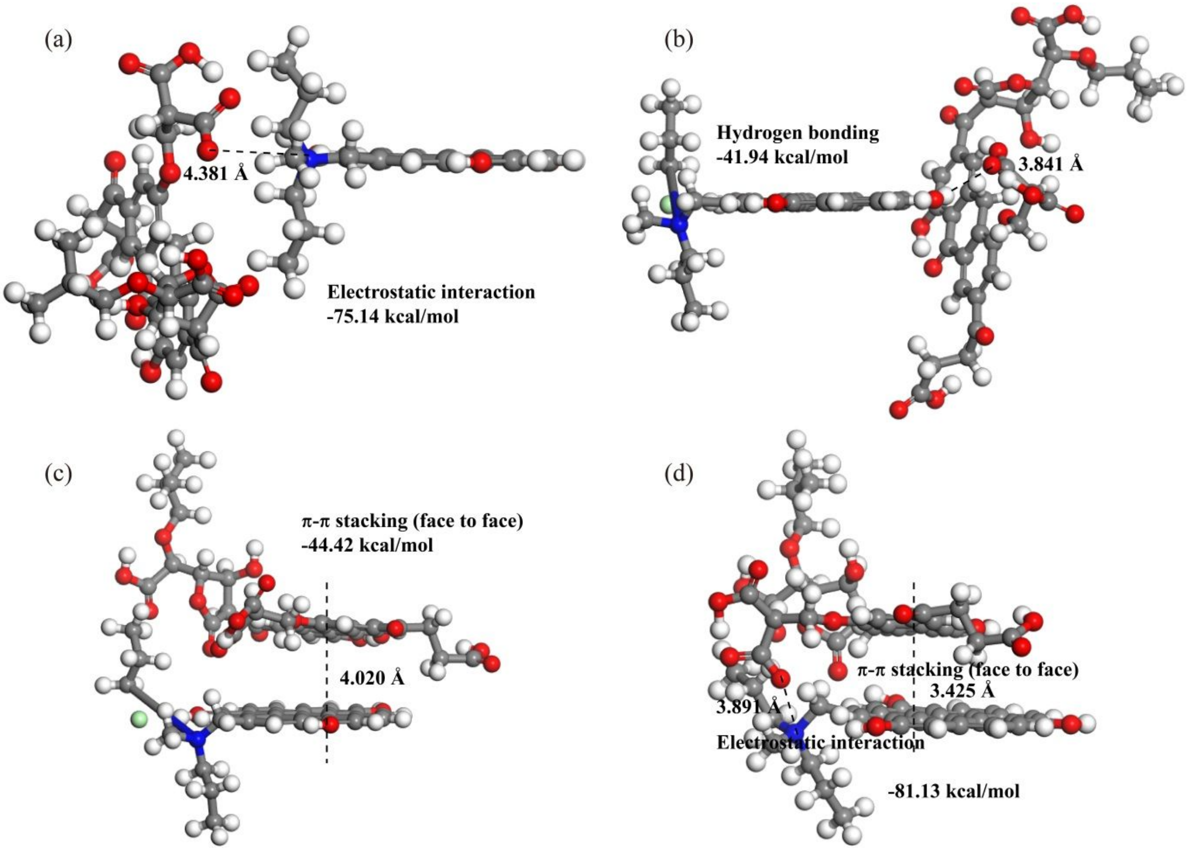

Figure S10 Optimized configurations of (a) electrostatic interaction, (b) hydrogen bonding, (c) π-π stacking (face to face), and (d) electrostatic interaction and π-π stacking (face to face) between $SRFA^-$ and PDDA-BC via DFT calculations at pH of approximately 6.

References

(1) Zeng, S.; Kan, E. Adsorption and Regeneration on Iron-Activated Biochar for Removal of Microcystin-Lr. *Chemosphere* **2021,** *273*, 129649.

(2) Song, H. J.; Gurav, R.; Bhatia, S. K.; Lee, E. B.; Kim, H. J.; Yang, Y.-H.; Kan, E.; Kim, H. H.; Lee, S. H.; Choi, Y.-K. Treatment of Microcystin-Lr Cyanotoxin Contaminated Water Using Kentucky Bluegrass-Derived Biochar. *J. Water Process Eng.* **2021,** *41*, 102054.

(3) Pavagadhi, S.; Tang, A. L.; Sathishkumar, M.; Loh, K. P.; Balasubramanian, R. Removal of Microcystin-Lr and Microcystin-Rr by Graphene Oxide: Adsorption and Kinetic Experiments. *Water Res.* **2013,** *47* (13), 4621-9.

(4) Park, J. A.; Jung, S. M.; Yi, I. G.; Choi, J. W.; Kim, S. B.; Lee, S. H. Adsorption of Microcystin-Lr on Mesoporous Carbons and Its Potential Use in Drinking Water Source. *Chemosphere* **2017,** *177*, 15-23.

(5) Park, J. A.; Kang, J. K.; Jung, S. M.; Choi, J. W.; Lee, S. H.; Yargeau, V.; Kim, S. B. Investigating Microcystin-Lr Adsorption Mechanisms on Mesoporous Carbon, Mesoporous Silica, and Their Amino-Functionalized Form: Surface Chemistry, Pore Structures, and Molecular Characteristics. *Chemosphere* **2020,** *247*, 125811.

(6) Huang, C.; Zhang, W.; Yan, Z.; Gao, J.; Liu, W.; Tong, P.; Zhang, L. Protonated Mesoporous Graphitic Carbon Nitride for Rapid and Highly Efficient Removal of Microcystins. *RSC Adv.* **2015,** *5* (56), 45368-45375.

(7) Park, J. A.; Jung, S. M.; Choi, J. W.; Kim, J. H.; Hong, S.; Lee, S. H. Mesoporous Carbon for Efficient Removal of Microcystin-Lr in Drinking Water Sources, Nak-Dong River, South Korea: Application to a Field-Scale Drinking Water Treatment Plant. *Chemosphere* **2018,** *193*, 883-891.

(8) Teng, W.; Wu, Z.; Feng, D.; Fan, J.; Wang, J.; Wei, H.; Song, M.; Zhao, D. Rapid and Efficient Removal of Microcystins by Ordered Mesoporous Silica. *Environ. Sci. Technol.* **2013,** *47* (15), 8633-41.

(9) Gao, Y. Q.; Gao, N. Y.; Deng, Y.; Gu, J. S.; Shen, Y. C.; Wang, S. X. Adsorption of Microcystin-Lr from Water with Iron Oxide Nanoparticles. *Water Environ. Res.* **2012,** *84* (7), 562-8.

(10) Yang, Y.; Hou, J.; Wang, P.; Wang, C.; Miao, L.; Ao, Y.; Wang, X.; Lv, B.; You, G.; Liu, Z.; Shao, Y. The Effects of Extracellular Polymeric Substances on Magnetic Iron Oxide Nanoparticles Stability and the Removal of Microcystin-Lr in Aqueous Environments. *Ecotoxicol. Environ. Saf.* **2018,** *148*, 89-96.

(11) Baziar, M.; Azari, A.; Karimaei, M.; Gupta, V. K.; Agarwal, S.; Sharafi, K.; Maroosi, M.; Shariatifar, N.; Dobaradaran, S. Mwcnt-Fe 3 O 4 as a Superior Adsorbent for Microcystins Lr Removal: Investigation on the Magnetic Adsorption Separation, Artificial Neural Network Modeling, and Genetic Algorithm Optimization. *J Mol. Liq.* **2017,** *241*, 102-113.

(12) Mashile, P. P.; Mpupa, A.; Nomngongo, P. N. Adsorptive Removal of Microcystin-Lr from Surface and Wastewater Using Tyre-Based Powdered Activated Carbon: Kinetics and Isotherms. *Toxicon : official journal of the International Society on Toxinology* **2018,** *145*, 25-31.

(13) Zhu, S.; Yin, D.; Gao, N.; Zhou, S.; Wang, Z.; Zhang, Z. Adsorption of Two Microcystins onto Activated Carbon: Equilibrium, Kinetic, and Influential Factors. *Desalination and Water Treatment* **2016,** *57* (50), 23666-23674.

(14) He, Y.; Wu, P.; Li, G.; Li, L.; Yi, J.; Wang, S.; Lu, S.; Ding, P.; Chen, C.; Pan, H. Optimization on Preparation of Fe(3)O(4)/Chitosan as Potential Matrix Material for the Removal of Microcystin-Lr and Its Evaluation of Adsorption Properties. *Int. J. Biol. Macromol.* **2020,** *156*, 1574-1583.

(15) Tian, X.; She, C.; Qi, Z.; Xu, X. Magnetic-Graphene Oxide Based Molecularly Imprinted Polymers for Selective Extraction of Microsystin-Lr Prior to the Determination by Hplc. *Microchem. J.* **2019,** *146*, 1126-1133.

(16) Xia, W.; Zhang, X.; Xu, L.; Wang, Y.; Lin, J.; Zou, R. Facile and Economical Synthesis of Metal–Organic Framework Mil-100(Al) Gels for High Efficiency Removal of Microcystin-Lr. *RSC Advances* **2013,** *3* (27), 11007.

(17) Zhang, H.; Zhu, G.; Jia, X.; Ding, Y.; Zhang, M.; Gao, Q.; Hu, C.; Xu, S. Removal of Microcystin-Lr from

Drinking Water Using a Bamboo-Based Charcoal Adsorbent Modified with Chitosan. *J. Environ. Sci. (China)* **2011,** *23* (12), 1983-8.
(18) He, X.; Pelaez, M.; Westrick, J. A.; O'Shea, K. E.; Hiskia, A.; Triantis, T.; Kaloudis, T.; Stefan, M. I.; de la Cruz, A. A.; Dionysiou, D. D. Efficient Removal of Microcystin-Lr by Uv-C/H2o2 in Synthetic and Natural Water Samples. *Water Res.* **2012,** *46* (5), 1501-10.
(19) Aleixo, L. M.; Godinho, O. E. S.; Da Costa, W. F. Potentiometric Study of Acid—Base Properties of Humic Acid Using Linear Functions for Treatment of Titration Data. *Anal. Chim. Acta* **1992,** *257* (1), 35-39.
